# A Novel Treatment for Glomerular Disease: Targeting the Activated Macrophage Folate Receptor with a Trojan Horse Therapy in Rats

**DOI:** 10.3390/cells10082113

**Published:** 2021-08-17

**Authors:** Gabriela E. Garcia, Yingjuan J. Lu, Luan D. Truong, Carlos A. Roncal-Jiménez, Makoto Miyazaki, Shinobu Miyazaki-Anzai, Gabriel Cara-Fuentes, Ana Andres-Hernando, Miguel Lanaspa, Richard J. Johnson, Christopher P. Leamon

**Affiliations:** 1 Department of Medicine, Division of Renal Diseases and Hypertension, University of Colorado Anschutz Medical Campus, Aurora, CO 80045, USA; Carlos.Roncal@cuanschutz.edu (C.A.R.-J.); makoto.Miyazaki@cuanschutz.edu (M.M.); Shinobu.Miyazaki-Anzai@cuanschutz.edu (S.M.-A.); Gabriel.Carafuentes@cuanschutz.edu (G.C.-F.); Ana.Andreshernando@cuanschutz.edu (A.A.-H.); Miguel.Lanaspagarcia@cuanschutz.edu (M.L.); Richard.Johnson@cuanschutz.edu (R.J.J.); 2 Endocyte, Inc., Novartis Institutes for Biomedical Research, West Lafayette, IN 47906, USA; june.lu@novartis.com (Y.J.L.); christopher.leamon@novartis.com (C.P.L.); 3 Department of Pathology, The Houston Methodist Hospital, Baylor College of Medicine, Houston, TX 77030, USA; LTruong@houstonmethodist.org

**Keywords:** macrophages, folate receptor, glomerulonephritis, inflammation, aminopterin, fibrosis

## Abstract

Since activated macrophages express a functional folate receptor β (FRβ), targeting this macrophage population with folate-linked drugs could increase selectivity to treat inflammatory diseases. Using a macrophage-mediated anti-glomerular basement membrane (anti-GBM) glomerulonephritis (GN) in WKY rats, we investigated the effect of a novel folic acid-aminopterin (AMT) conjugate (EC2319) designed to intracellularly deliver AMT via the FR. We found that treatment with EC2319 significantly attenuated kidney injury and preserved renal function. Kidney protection with EC2319 was blocked by a folate competitor, indicating that its mechanism of action was specifically FRβ-mediated. Notably, treatment with methotrexate (MTX), another folic acid antagonist related to AMT, did not protect from kidney damage. EC2319 reduced glomerular and interstitial macrophage infiltration and decreased M1 macrophage recruitment but not M2 macrophages. The expression of CCL2 and the pro-fibrotic cytokine TGF-β were also reduced in nephritic glomeruli with EC2319 treatment. In EC2319-treated rats, there was a significant decrease in the deposition of collagens. In nephritic kidneys, FRβ was expressed on periglomerular macrophages and macrophages present in the crescents, but its expression was not observed in normal kidneys. These data indicate that selectively targeting the activated macrophage population could represent a novel means for treating anti-GBM GN and other acute crescentic glomerulonephritis.

## 1. Introduction

Inflammation is a key factor in the induction and progression of glomerulonephritis (GN) and other kidney diseases [1,2]. One of the key inflammatory cells is the macrophage, and especially the classically activated or M1 macrophage phenotype. These cells are present in most human kidney diseases, not simply glomerulonephritis. For example, macrophages are present in diabetic nephropathy, polycystic kidney disease, kidney allograft rejection, chronic allograft nephropathy, and acute kidney injury. In human biopsy studies, glomerular or interstitial macrophages correlates numerically with poor outcomes. In addition, activated macrophage correlates with the worst course of the disease [1,3].

The importance of the macrophage has been demonstrated in a variety of ways, such as specifically depleting macrophages in various experimental models [4,5]. In addition, inactivating macrophages or inhibiting chemokines mediating-macrophage recruitment attenuates kidney injury [6,7,8,9]. Direct evidence that macrophages induce kidney injury has been demonstrated using the adoptive transfer of macrophages in the anti-glomerular basement membrane (GBM) GN model. Moreover, there was also a positive correlation between the number of transferred glomerular macrophages and the severity of the kidney damage [10].

In chronic disease, activated macrophages continue to release cytokines/chemokines, digestive enzymes, prostaglandins, and reactive oxygen species, which can aggravate or accelerate damage to the normal tissues. The final common pathway of chronic kidney disease that leads to kidney replacement therapy appears to be driven, at least in part, by chronically activated macrophages [1,11].

Given these findings, a drug that could selectively target the activated macrophage population could represent a novel means for targeting chronic inflammatory diseases. Strategies to target macrophages as new therapies to treat inflammatory diseases in humans are emerging. For example, recombinant serum amyloid P binds to macrophage FcγRs and inhibits macrophage activation in part by the release of IL-10 [12]. Inhibition of macrophage recruitment by blocking chemokines receptors has been used as an alternative therapy. However, problems faced with targeting individual components of monocyte/macrophage recruitment have been the redundancy of chemokines/chemokines receptors and the potential inhibition of innate cellular immune response to pathogens [1,13].

For reasons not fully understood, a functional folate receptor (FRβ) is expressed on activated macrophages but not resting macrophages and is considered as a biomarker for macrophage activation. FRβ displays nanomolar affinities for folic acid (FA), and cellular uptake of FA and FA-containing ligands is equivalent to the level of FR expression [14,15,16]. This finding has led to the hypothesis that FRβ-expressing macrophages can be targeted with FA-linked drugs without promoting drug uptake by nonactivated macrophages.

Aminopterin (AMT) is a highly active antifolate and antimetabolite that has strong anti-inflammatory effects in rheumatoid arthritis and psoriasis. However, because of its toxicity, it was discontinued in favor of the more tolerable agent, methotrexate (MTX). AMT has superior potency and pharmacological properties over MTX; it is 40-fold and 20-fold more potent in murine models of air-pouch inflammation and arthritis than MTX. These features have resulted in renewed interest in AMT clinical development for oncology and inflammatory diseases (https://clinicaltrials.gov/ct2/show/NCT03431974, accessed on 15 January 2021). Unfortunately, the toxicity of both AMT and MTX is from indiscriminate uptake by normal cells via the reduced folate carrier, that it is ubiquitously expressed, and the low pH-dependent intestinal proton-coupled folate transporter [17,18,19].

**Figure 1:**
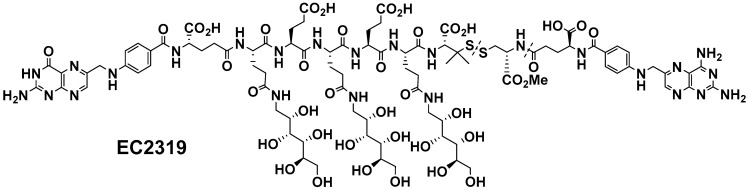
EC2319 chemical structure.

Previously, we developed a highly specific FA-conjugated AMT therapy for intracellular delivery of AMT via the FR. This early compound (EC0746) demonstrated two independent mechanisms of action in in vitro cells models: an anti-proliferative effect against RAW264.7 cells and an anti-inflammatory effect against rat peritoneal macrophages. EC0746 inhibited the release of several cytokines and chemokines, including IL-1β, TNF-α, CCL3/MIP-1α, and MIG/CXCL9 in fully activated peritoneal macrophages with LPS and IFNγ [20]. Both anti-proliferative and anti-inflammatory effects were prevented in the presence of an excess of folic acid, indicating an FR-specific mode of action. EC0746 also suppressed active inflammation targeting FRβ-expressing macrophages in adjuvant arthritis, autoimmune uveitis, and autoimmune encephalomyelitis [18,20]. For pre-clinical translation, EC2319, a different compound with the novel, improved linker design, was subsequently developed (Figure 1). In the present study, we investigated the effect of EC2319 in the progression of kidney injury in the anti-GBM GN model.

## 2. Materials and Methods

### 2.1. EC2319 Modular Design

The EC2319 chemical structure (Figure 1) consists of four functional components: the FR-targeting moiety FA, the drug moiety AMT, a saccharo-amino acid peptide-based spacer (designed to reduce conjugate liver clearance), and an l-cysteine methyl ester linker with a protected disulfide bond (to remain largely stable in the circulation but to be cleaved within the endosomal structures). EC2319 displays high FR-binding affinity and specificity in vitro and in vivo. Details in EC2319 synthesis and characterization are included in a separate manuscript [21].

### 2.2. Induction, Treatment, and Analysis of Accelerated Anti-GBM GN

Animal studies were approved by the IACUC at the University of Colorado Denver. Male WKY rats (Charles River, Wilmington, MA, USA) weighing 180–200 g were maintained on a low folate diet (Envigo Teklad, Madison, WI, USA) for 10 days to decrease folate closer to human levels [22]. Then, rats received an intravenous injection of 25 µL/100 g body weight of anti-GBM antibody to induce anti-GBM GN as described [6,23,24,25]. Treatment with EC2319 was started during the acute inflammatory phase of the disease; EC2319, 750 nmol/kg s.c., biweekly, was given 8 h after the injection of anti-GBM antibody (maximum glomerular deposition of IgG occurs one hour after an injection of anti-GBM antibody [26]) after infiltration of CD8^+^ cells and macrophages were already started [6,27]. Urine protein excretion was measured on timed 24 h specimens at days 9 by the sulfosalicylic method [8]. Rats were euthanized on day 10 after the induction of the disease to collect the kidney, spleen, and blood. Serum creatinine levels were determined by liquid chromatography-tandem mass spectrometry [28].

To demonstrate in vivo target specificity of EC2319, a benign FA-containing competitor was used in 300-fold molar excess to block the activity of EC2319. In this study, EC2319 was used at a dose of 575 nmol/kg s.c., biweekly. In addition, the effect of EC2319 was compared with MTX, which can be used to treat anti-GBM GN in humans [29,30]. MTX was given either orally at 2067 mg/kg once a week (MTX Wk), which is close to the average weekly human dose for rheumatoid arthritis and anti-GBM GN (15–25 mg/kg [29,30,31,32]) or orally at equimolar basis to EC2319 (575 nmol/kg, biweekly, MTX 3D). Treatments were started at the acute phase of the disease, and rats were euthanized on day 10 after the induction of anti-GBM GN as described above.

### 2.3. mRNA Expression of Chemokines and Cytokines

MDC/CCL22 (400 bp), RANTES/CCL5 (246 bp), MIP-1α/CCL3 (284 bp), fractalkine/CX3CL1 (420 bp), MIP-3β/CCL19 (380 bp), MIP-1β/CCL4 (210 bp), MCP-1/CCL2 (239 bp), and L-32 (92 bp) riboprobes were generated by PCR reaction using cDNA templates as described [6,8,33]. rCK1 (BD Pharmingen, San Diego, CA, USA) was used to investigate cytokine expression. Glomeruli were prepared by sequential sieving, and total RNA was isolated from glomeruli [34,35]. A total of 3 µg of total RNA from each sample were used in an RNase protection assay using the Torrey Pines Biolabs kit (Secaucus, NJ, USA) as described [35,36,37,38]. Phosphoimage quantitation was performed using the PhosphorImager SI scanning instrument and ImageQuaNT software (Molecular Dynamics, Sunnyvale, CA, USA) [36,39,40].

### 2.4. Morphological Analysis, Immunohistochemical Phenotyping, and Quantitation of Leukocytes

Kidney samples fixed in formalin or methanol-Carnoy fixative solution were embedded in paraffin. Sections of 2–3 µm were stained with periodic acid-Schiff (PAS) reagent to assess glomerular hypercellularity, necrotizing lesions, and formation of glomerular crescents (crescentic glomeruli per 100 glomeruli was calculated and expressed as a percentage). Infiltrating leukocytes were immunohistochemically stained for ED1^+^ (catalog MCA341R AbD Serotec), CD169^+^, clone ED3, a marker for MI macrophages (catalog MCA343GA, AbD Serotec), and CD163^+^, clone ED2, a marker for M2 macrophages (catalog MCA342R, AbD Serotec) infiltrates. Slides were reacted with mouse anti-rat ED1, anti-rat CD169, and anti-rat CD163 and peroxidase-coupled anti-mouse IgG second antibodies (catalog P044701-2, Agilent/Dako) as described [6,24,35]. Positively stained cells per 100 glomeruli were counted and expressed per glomerular section. All quantitative morphological analyses were performed in a blinded fashion.

### 2.5. Immunohistochemistry of Collagen

Paraffin sections of methanol-Carnoy fixed tissue were stained for collagens I, III, and IV with specific antibodies (catalog 1310-01, 1330-01, and 1340-01, Southern Biotech, Birmingham, AL, USA, respectively). The secondary antibodies consisted of peroxidase-coupled rabbit anti-goat IgG (catalog P044901-2, Agilent/Dako, Santa Clara, CA, USA). Histological morphometry was performed using a ScanScope digital scanner (Aperio Technologies, Inc., Vista, CA, USA), and results were expressed as mean ± SEM% area.

### 2.6. Immunohistochemistry of FRβ

Kidney sections were stained with a specific monoclonal mouse anti-rat FRβ antibody (1:100, a gift from Takami Matsuyama, Kagoshima University, Kagoshima, Japan [41]) and horseradish peroxidase-conjugated anti-mouse Ig second antibody (Goat anti-mouse IgM, catalog NB7497, Novus, Centennial, CO, USA).

### 2.7. Immunofluorescence Staining of FRβ and Macrophages

Frozen sections were stained with mouse anti-rat FRβ antibody and anti-mouse IgM AlexaFluor 488 (green, catalog A-21042, Thermo Fisher Scientific, Waltham, MA, USA). Macrophages were stained with anti-ED1 and anti-mouse IgG AlexaFluor 594 (red, catalog A-21203). Labeled tissues were visualized using confocal microscopy (Olympus FV1000).

### 2.8. Circulating Ab and Glomerular IgG Deposition

Rat anti-rabbit IgG Ab titers were measured by enzyme-linked immunosorbent assay using sera collected at day 10 after the induction of nephritis as described [27,33]. Bound rat IgG was detected using peroxidase-conjugated anti-rat IgG (catalog P0450, Agilent/Dako) at 1:1000 and absorbance reading at 450 nm. Normal sera served as a negative control. IgG deposition was determined in kidney frozen sections using FITC-labeled anti-rabbit IgG (catalog F020502-2, Agilent/Dako) or anti-rat IgG (catalog 3030-02, Southern Biotech) at different dilutions as described [6,33]. Immunofluorescence images were analyzed by Image J software version 1.48v (NIH, Bethesda, MD, USA) [8].

### 2.9. Statistics

Statistical analyses were performed using the one-way ANOVA with multiple pairwise comparisons with the Bonferroni adjustment for multiple hypothesis testing. Student’s *t*-test (Mann–Whitney U-test) was used to compare mean values between two experimental groups. Data are reported as mean values ± SEM. Values of *p* < 0.05 were considered statistically significant.

## 3. Results

### 3.1. EC2319 Protects from Kidney Injury in Anti-GBM GN

**Figure 2:**
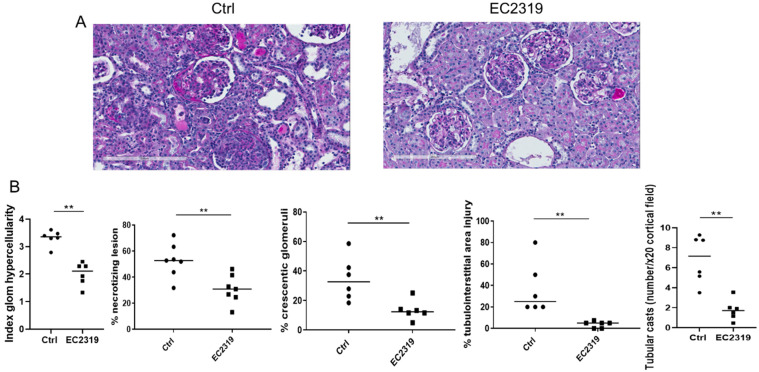
EC2319 attenuates kidney injury: (**A**) Periodic acid-Schiff (PAS) staining of kidney sections of WKY rats treated with vehicle or EC2319. Nephritic kidneys from vehicle-treated rats had severe lesions that were reduced in the EC2319 treated group. (**B**) Quantitation of kidney injury in control rats and EC2319-treated rats with anti-GBM GN. Results were sampled from six rats per group and expressed as mean ± SEM. ** *p* < 0.01. PAS staining, original magnification ×400. • Control, ■ EC2319.

In control rats, anti-GBM GN led to severe glomerular hypercellularity, necrotizing lesions, and crescentic formation. In contrast, the degree of kidney injury was markedly reduced in rats treated with EC2319. The index of hypercellularity (3.3 ± 0.062 vs. 1.9 ± 0.073, *p* < 0.01), necrotizing lesions (52.7% ± 5.3% vs. 30.8% ± 4.4%, *p* < 0.01) and crescent formation (34.6% ± 5.5% vs. 13.4% ± 2.4%, *p* < 0.01) were reduced by 52.5%, 41.6%, and 61.3%, respectively (Figure 2A,B). Treatment with EC2319 also decreased the tubulointerstitial (TIN) injury (36.7% ± 9.12% vs. 3.75% ± 1.22%, *p* < 0.01) and the number of tubular casts, an indicator for chronic TIN injury, (6.84 ± 2.75 vs. 1.77 ± 1.3, *p* < 0.01) (Figure 2A,B).

**Figure 3:**
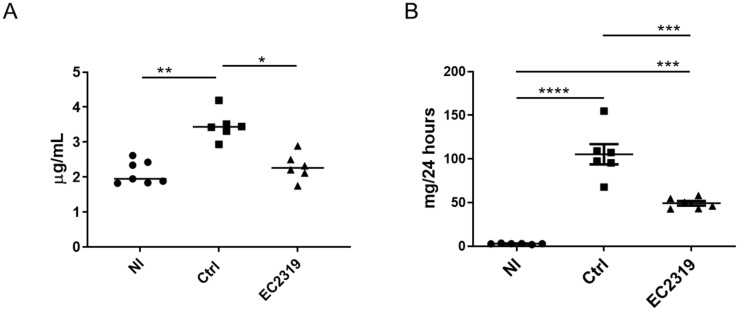
Kidney function is preserved, and proteinuria is decreased with EC2319 in anti-GBM GN: (**A**) Serum creatinine levels (µg/mL) in WKY rats treated with vehicle or EC2319. Serum creatinine in the EC2319-treated group was not different from normal controls. (**B**) EC2319 significantly reduced proteinuria (mg per 24 h) compared to the control group. Results were sampled from six rats per group and expressed as mean ± SEM. * *p* < 0.05, ** *p* < 0.01, *** *p* < 0.001, **** *p* < 0.0001. • Normal, ■ Control, ▲ EC2319.

As a result of attenuation of kidney damage in EC2319-treated rats, kidney function was preserved compared to the control rats (Normal, Nl 2.1 ± 0.15 µg/mL, Ctrl 3.37 ± 0.16 µg/mL, EC2319 2.2 ± 0.145 µg/mL) (Figure 3A). EC2319 also reduced proteinuria compared to the control group (Ctrl 109.25 ± 11.8 vs. 49.17 ± 2.6 mg/24 h, *p* < 0.001) (Figure 3B). These results indicate that treatment with EC2319 at the acute phase of anti-GBM GN confers kidney protection from damage.

#### 3.1.1. Kidney Protection in GN by EC2319 Is Associated with Reduced Macrophage Infiltration

**Figure 4:**
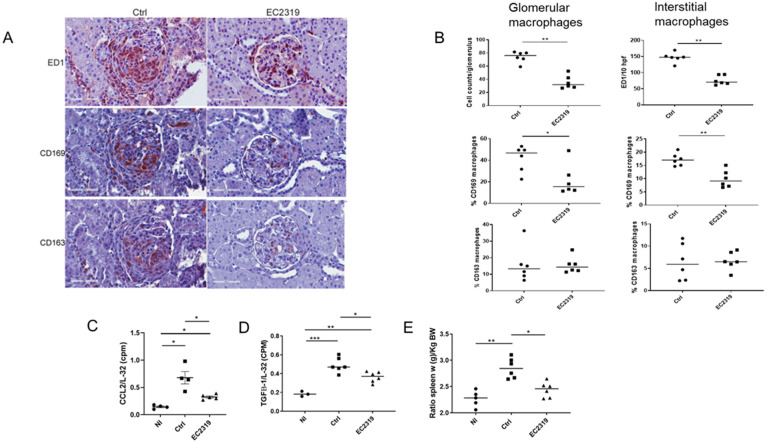
Anti-inflammatory effect of EC2319. (**A**) Immunohistochemistry stained for ED1^+^ monocyte/macrophages, CD163^+^, and CD 169^+^ infiltrates of kidney sections of rats with anti-GBM GN. (**B**) Quantification of ED1^+^, CD163^+^, and CD169^+^ infiltration. In rats treated with EC2319, the percentage of glomerular and interstitial M1 macrophages was significantly reduced without affecting the M2 macrophage phenotype. (**C**,**D**) RNase protection assay of cytokines and chemokines expressed in the glomeruli of anti-GBM glomerulonephritis in WKY rats. EC2319 decreases the expression of TGF-β and MCP-1/CCL2. (**E**). Spleen weight in control rats and EC2319 rats. Splenomegaly was prevented in the EC2319-treated group. At least sixty glomeruli per section were counted. For RPA, the data are presented as a ratio of the cpm for the specific mRNA/L-32 mRNA to ensure a constant quantity of RNA in each sample. Spleen weight is expressed as relative to body weight. Results were sampled from 6 control and 6 treated rats with anti-GBM GN and expressed as mean ± SEM. For RPA, 3 normal WKY rats were included. * *p* < 0.05, ** *p* < 0.01, *** *p* < 0.001. In (**B**): • Control, ■ EC2319. In (**C**–**E**): • Normal, ■ Control, ▲ EC2319.

We next examined the effect of EC2319 on macrophage recruitment. As shown in Figure 4, there was a prominent accumulation of ED1^+^ macrophages in the glomeruli and interstitium of untreated rats with GN. EC2319 treatment attenuated the infiltration of these macrophages by 52% in the glomeruli (74 ± 3.1 vs. 35.5 ± 3.75, *p* < 0.01) and 48.1% in the interstitium (147.03 ± 4.19 vs. 76.3 ± 3.36, *p* < 0.01) compared with the untreated group (Figure 4A,B). CD8^+^ cells were not detected at this time of the disease (their peak influx is at day 3 after the induction of the disease and decreased thereafter [8,27,35]).

Since we have previously demonstrated EC2319 targets activated macrophages, we next investigated if the treatment with EC2319 in GN could affect M1 (proinflammatory) and M2 (anti-inflammatory reparative) macrophage populations. Just as ED1^+^ macrophage infiltration was reduced in the EC2319-treated group, M1 macrophage phenotype (CD169^+^ cells) was also decreased in this group compared to the control group 30.8 ± 3.55 vs. 8.55 ± 1.72, *p* < 0.05. While M2 macrophages (CD163^+^ cells) were also reduced in the EC2319 group, it was not statistically significant (11.89 ± 3.3 vs. 5.4 ± 0.65 NS). Moreover, 21.7% ± 5.39% of the infiltrating macrophages in the EC2319-treated group was the M1 phenotype compared to 41.7% ± 4.46% in the control group (*p* < 0.05). However, there was no difference in the percentage of M2 macrophage phenotype between the EC2319 group and the control group (15.53 ± 1.85 vs. 15.7 ± 4 vs. NS) (Figure 4A,B). In the interstitium, although not all macrophages were recognized by CD169 and CD163, the percentage of M1 macrophages was significantly reduced in the EC2319-treated group compared to the controls (17.2 ± 0.87 vs. 9.87 ± 1.2, *p* < 0.01). However, there was no difference in the percentage of M2 interstitial macrophages between the treated group and control groups (6.68 ± 0.75 vs. 6.55 ± 1.5 NS) (Figure 4A,B). These data denote that EC2319 primarily decreases M1 macrophages with a nonsignificant effect on the M2 macrophage phenotype.

#### 3.1.2. EC2319 Modulates the Expression of Chemokines and Cytokines

We next determine if the effect of EC2319 to reduce macrophage infiltration was associated with the suppression of cytokines/chemokines. We found EC2319 significantly decreased the expression of the pro-fibrotic cytokine TGF-β and CCL2/MCP-1, a chemokine that is not only produced by activated macrophages but also plays a key role in the infiltration of these cells in anti-GBM GN [6,7,8,42,43] (Figure 4C,D).

Similarly, the anti-inflammatory activity of EC2319 was demonstrated by the reduction in splenomegaly caused by systemic inflammation. As shown in Figure 4E, the spleen weight in EC2319-treated rats was not different from normal rats, but splenomegaly was observed in control rats (Normal, Nl 2.44 ± 0.52 vs. Ctrl 2.85 ± 0.07, *p* < 0.01).

### 3.2. EC2319 Decreases Collagen Deposition

**Figure 5:**
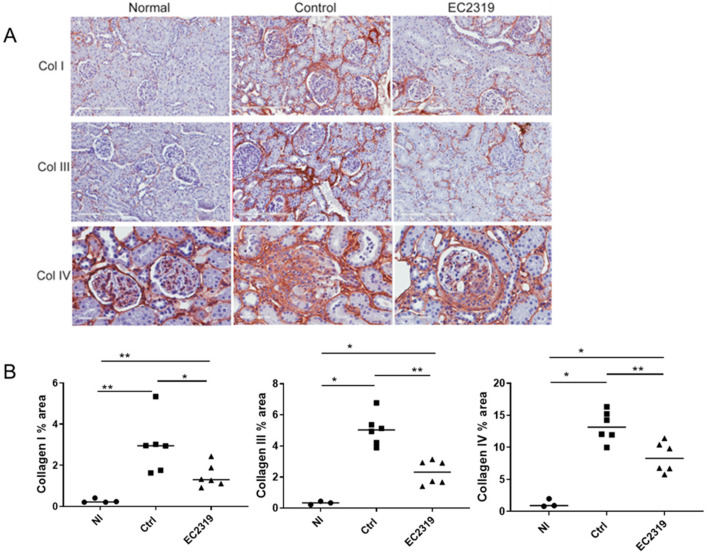
Collagen deposition is decreased in rats with anti-GBM GN and treated with EC2319. (**A**) Collagen I and collagen III expression was reduced in the interstitium, and collagen IV deposition was decreased in the glomeruli from nephritic kidneys in EC2319-treated rats. (**B**) Morphometric analysis of collagen I, collagen III, and collagen IV. Each data point represents sections sampled from 3 normal WKY rats and 6 control and 6 treated rats. Results are expressed as mean ± SEM percent (%) area. * *p* < 0.05, ** *p* < 0.01. Collagens staining I and III, original magnification × 200, and collagen IV original magnification ×400. • Normal, ■ Control, ▲ EC2319.

To investigate if EC2319 could prevent progressive kidney injury, we examined the deposition of collagen. In control rats, increased deposition of collagen I and collagen III was observed compared to EC2319-treated rats (2.94% ± 0.49% area vs. 1.48% ± 0.21% area, *p* < 0.05 and 5.05% ± 0.37% area vs. 2.29% ± 0.28% area, *p* < 0.01, respectively) (Figure 5A,B). In addition, the expression of collagen IV, an important component of the glomerular extracellular matrix, was also enhanced in the nephritic glomeruli of controls rats (13.29% ± 0.88% area vs. 8.47% ± 0.88% area, *p* < 0.01) (Figure 5A,B). These data suggest that EC2319 treatment protects from developing progressive GN.

### 3.3. FRβ Is Expressed in Nephritic Glomeruli

**Figure 6:**
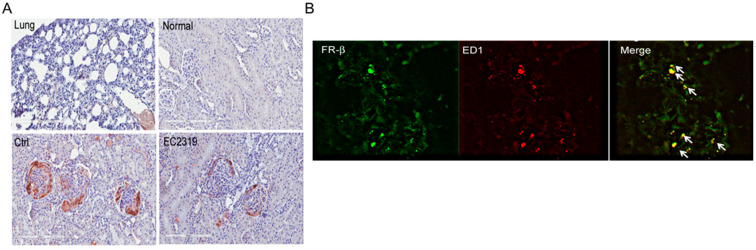
FRβ is expressed in the glomeruli from rats with anti-GBM GN: (**A**) FRβ staining. Lung was used as a negative control. The normal kidney does not express FRβ; in the control-treated group, nephritic glomeruli show markedly expression of FRβ that is decreased in rats treated with EC2319. (**B**) Double staining for FRβ (green) and ED1+ monocyte/macrophages (red). FRβ is expressed in periglomerular macrophages and macrophages localized in the crescents (white arrows).

As shown in Figure 6, the high-affinity folate receptor, FRβ, was expressed in the glomeruli, mainly periglomerular and crescents, of rats with GN but not in normal kidneys or in the lung (Figure 6A). The expression of FRβ was reduced in rats treated with EC2319. The lung was used as a negative control to demonstrate that the antibody does not react with FRα that is preferentially expressed on the apical surface of the epithelia. To determine the localization of FRβ double immunofluorescence staining for FRβ and ED1 was performed. We found ED1^+^ macrophages, especially periglomerular and in the crescents, express FRβ (Figure 6B). These data indicate FRβ is expressed in macrophages in anti-GBM GN, and treatment with EC2319 reduces its expression probably because of decreased infiltration of activated macrophages.

### 3.4. Blocking EC2319 Binding to Folate Receptor Prevents Its Protective Effect in GN

**Figure 7:**
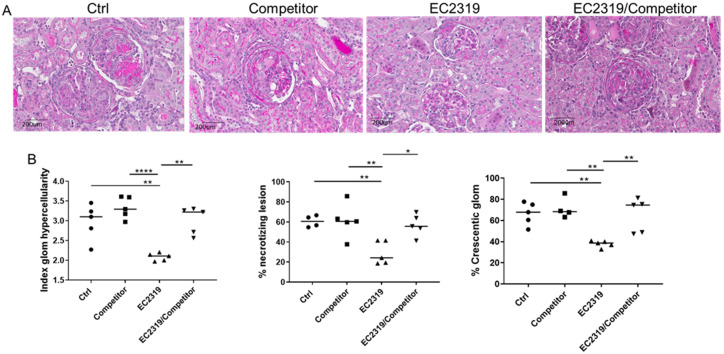
The protective effect of EC2319 in GN is reversed by blocking its binding to folate receptors. (**A**) Periodic acid-Schiff (PAS) staining of kidney sections of WKY rats treated with vehicle, a folate-containing competitor, EC2319, and EC2319 and FA competitor (EC2319/Competitor). The FA competitor does not affect kidney injury in GN. EC2319 reduces kidney damage; this effect is reversed with the simultaneous treatment with the competitor. (**B**) Quantitation of kidney injury in control rats, Competitor, EC2319, and EC2319/competitor-treated rats with anti-GBM GN. Each data point represents sections sampled from five rats and is expressed as mean ± SEM. * *p* < 0.05, ** *p* < 0.01, **** *p* < 0.0001. PAS staining, original magnification × 400. • Control, ■ Competitor, ▲ EC2319, ▼ EC2319/Competitor.

To confirm the in vivo target specificity of EC2319, a competitive study was performed using a benign FA-containing competitor (a high-affinity water-soluble FA-peptide conjugate) to block FR binding of EC2319 [20]. Four groups of rats with anti-GBM GN were studied: control rats, rats treated with EC2319 alone (575 nmol/kg), rats that received EC2319 (575 nmol/kg) plus 300-fold molar excess of the FA competitor, and rats treated with FA competitor alone. As shown in Figure 7A,B, EC2319 protected from kidney injury with significant reduction in glomerular hypercellularity (2.97 ± 0.18 vs. 2.08 ± 0.039 *p* < 0.01), necrotizing lesion (60.65 ± 2.27 vs. 29.04 ± 4.64 *p* < 0.01), and crescent formation (66.54% ± 4.31% vs. 37.88% ± 1.28%, *p* < 0.01) compared with the control group (Figure 7A,B). The innocuous FA competitor did not affect kidney damage since parameters of glomerular injury were similar to those in the control group; however, treatment with EC2319 plus the FA competitor reversed the protective effect of EC2319 (Figure 7A,B). These data indicate that EC2319 attenuation of the kidney injury in GN is predominantly FR-mediated.

### 3.5. MTX Is Unable to Protect from Kidney Injury in Anti-GBM GN

**Figure 8:**
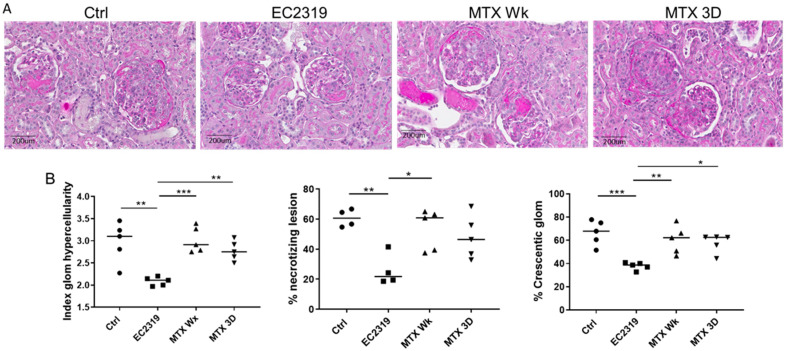
MTX does not protect from kidney injury in anti-GBM GN: (**A**) PAS staining of kidney sections of WKY rats with anti-GBM GN and treated with vehicle, EC2319, MTX once a week (MTX Wk), and MTX at equimolar basis to EC2319 (MTX 3D). (**B**) Quantitation of kidney injury in control rats, rats treated with EC2319, MTX Wk and MTX 3D. In control rats, severe glomerular hypercellularity, necrotizing lesion, and crescent formation were observed. In rats treated with EC2319, the glomerular injury is significantly reduced; however, MTX (MTX Wk and MTX 3D) is unable to attenuate kidney damage. Results were sampled from five rats per group and expressed as mean ± SEM. * *p* < 0.05, ** *p* < 0.01, *** *p* < 0.001. • Control, ■ EC2319, ▲ MTX Wk, ▼ MTX 3D.

We next compared the effect of EC2319 against MTX given either orally at 2067 mg/kg once a week (equivalent to an average human dose for anti-GBM GN, MTX Wk [29]) or at an equimolar basis to EC2319 (575 nmol/kg, MTX 3D). Neither MTX treatments protected from kidney damage in GN (Figure 8A,B). In contrast, EC2319 improved overall glomerular scores (Figure 8A,B).

### 3.6. EC2319 and MTX Decrease Antigen-Specific Humoral Immune Response

**Figure 9:**
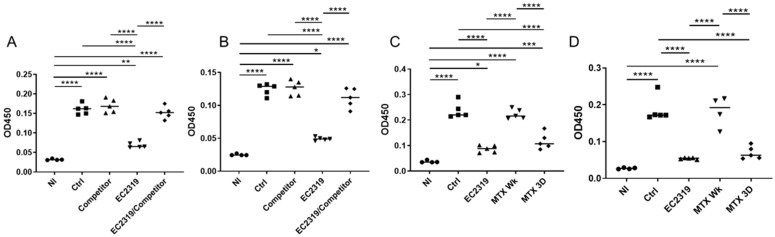
Systemic antigen-specific humoral immune response is attenuated by EC2319 and MTX: (**A**–**D**) circulating titers of total rat anti-rabbit IgG at serum dilution of 1:20 (**A**,**C**) and 1:100 (**B**,**D**) are decreased by EC2319 and MTX Wk but not by MTX 3D. The reduction in anti-rabbit IgG levels by EC2319 is prevented in rats treated with EC2319 plus FA competitor (**A**,**B**). * *p* < 0.05, ** *p* < 0.01, *** *p* < 0.001, **** *p* < 0.0001. For (**A**,**B**): • Normal, ■ Control, ▲ Competitor, ▼ EC2319, ♦ EC2319/Competitor. For (**C**,**D**): • Normal, ■ Control, ▲ EC2319, ▼ MTX Wk, ♦ MTX3D.

To analyze if the systemic humoral immune response could be affected by EC2319 and MTX, we measured serum antigen-specific total rat anti-rabbit IgG by ELISA. Total rat anti-rabbit globulin IgG levels were significantly reduced by EC2319 and MTX matching EC2319 dose (MTX 3D) at different dilutions (Figure 9). The decrease in total rat anti-rabbit IgG levels was similar between both groups. However, MTX administrated orally once a week (MTX Wk) did not affect serum anti-rabbit globulin IgG levels (Figure 9C,D). Interestingly, reduction in circulating anti-rabbit globulin IgG by EC2319 was prevented in rats treated with EC2319 plus the FA competitor (Figure 9A,B).

### 3.7. EC2319 and MTX Reduce Glomerular Rat Igg Deposition

**Figure 10:**
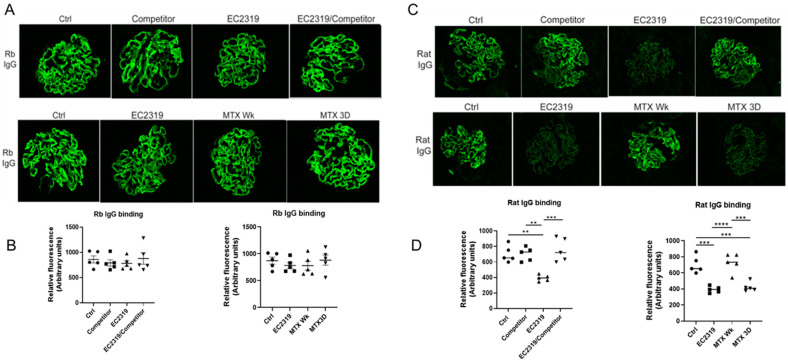
Glomerular rabbit IgG deposition is not affected by treatment with EC2319, EC2319/FA competitor, or MTX; however, glomerular rat IgG deposition is decreased in EC2319- and MTX Wk-treated rats with GN. (**A**) Immunofluorescence staining of rabbit IgG reveals rabbit IgG along the capillary walls of glomeruli in a linear pattern with no discernible difference in the intensity between the groups. (**B**) Quantification intensity of rabbit IgG immunofluorescence staining. (**C**) Immunofluorescence staining of rat IgG shows a decrease in the intensity within the glomeruli in rats treated with EC2319 and MTX Wk. (**D**) Quantification of rat IgG fluorescence intensity. ** *p* < 0.01, *** *p* < 0.001, **** *p* < 0.000. For (**B**,**D** **left** panel): • Control, ■ Competitor, ▲ EC2319, ▼ EC2319/Competitor. For (**B**,**D** **right** panel): • Control, ■ EC2319, ▲ MTX Wk, ▼ MTX3D.

In the glomeruli, no difference in the intensity and distribution of rabbit IgG staining was observed between the groups (Figure 10A,B). However, decreased deposition of rat IgG within the glomeruli was observed in rats treated with EC2319 and with MTX matching EC2319 dose (MTX 3D) (Figure 10C,D). The decreased in rat IgG deposits was similar in both groups. Since the FA competitor reversed the decrease in circulating levels of rat IgG in EC2319-treated rats, deposition of rat IgG in the EC2319/FA competitor-treated group was not different from that of the control group (Figure 10C,D).

These data indicate none of the treatments affects the induction of anti-GBM GN, but EC2319 and MTX3D decrease systemic IgG and rat IgG deposits within the glomeruli.

## 4. Discussion

Anti-GBM GN is a life-threatening disease in which inflammation in the glomerulus leads to rapidly progressive GN. Unfortunately, current treatment options remain limited, with costly, unspecific, and side-effect-prone immunosuppressive therapies.

Despite treatment, less than one-third of the patients with anti-GBM GN survive with a preserved kidney function after 6 months of follow-up [44]. More recently, in a French nationwide multicenter cohort study, it was reported that at 3 months after the diagnostic of anti-GBM GN, 46% of patients developed end-stage kidney disease [45]. In a multicenter worldwide study, the 5-year kidney survival in patients with anti-GBM GN was only 34%. Although kidney survival has increased two-fold since 2007, mainly because of earlier detection and intensive therapy (plasmapheresis, corticosteroids, and other cytotoxic drugs [46]), it is clear that safe, more specific, and well-tolerated innovative treatments are needed to treat anti-GBM GN.

In patients with this crescentic GN, macrophage infiltration into glomeruli was demonstrated more than 40 years ago [47]. There is a clear association between macrophage accumulation and the development of progressive kidney injury [3]. An early study using anti-rabbit macrophage serum to block macrophage infiltration was showed a marked reduction in kidney injury without affecting the underlying immunological mechanism of the disease [5]. Moreover, the reduction in macrophages in the established phase of crescentic GN prevents the progression of kidney damage [4]. Consequently, strategies that selectively block activated monocytes/macrophages at the site of inflammation are highly attractive for the treatment of anti-GBM GN.

MTX is not a widely used treatment option for the anti-GBM disease. However, according to the KIDGO-GN guideline, MTX is indicated as maintenance therapy in human anti-GBM GN, although it is also used as induction therapy [29,30]. AMT is the precursor of MTX and was the first antifolate used to treat inflammatory disorders. AMT is more potent than MTX; however, it is also more toxic. Previously, EC0746, an early AMT conjugate, was found to be approximately 40-fold less toxic than unmodified AMT because of its selective uptake by FRβ expressing inflammatory cells [20].

In this study, we evaluated a novel EC2319 construct that uses the high-affinity FA ligand to facilitate the specific delivery of AMT to FRβ expressing cells in accelerated anti-GBM GN in WKY rats. This model is comparable with anti-GBM antibody-mediated crescentic GN in humans and progresses rapidly to lethal kidney failure by a macrophage-mediated mechanism. In this severe anti-GBM GN model, EC2319 attenuates inflammation and infiltration of M1 macrophages into the kidney, significantly reduces kidney injury, and effectively inhibits fibrotic progression.

This protective effect appears to be, at least in part, FR-mediated since a benign folate ligand efficiently blocked its overall effect by competing for FR-binding sites. Treatment was also superior to oral administration of a general folate antagonist (MTX), documenting that specific targeting of FRβ^+^-activated macrophages is a possible new means of blocking glomerular injury.

In addition, in rats with anti-GBM GN, we have detected the presence of activated monocytes that were significantly decreased with EC2319 treatment. This effect was FR-specific and blocked in rats treated with EC2319 plus the FA competitor. No reduction in activated monocytes was seen in rats with anti-GBM GN treated with MTX given either orally (2067 mg/kg once a week, MTX Wk) or at equimolar basis to EC2319 (MTX 3D) [21].

Interestingly, we also observed EC2319 reduced circulating levels of antigen-specific IgG (rat anti-rabbit globulin IgG) and decreased rat IgG deposition within glomeruli. As EC2319 effect on the humoral immune response was prevented with an excess of the FA-containing competitor, this phenomenon could not be explained by the immunosuppressive effect of free AMT or its activate metabolites released by EC2319 in the circulation (~9% and 3% in female Lewis rats [21]). By chemical design, neither EC2319 nor the FA competitor can release free FA for cellular uptake by the reduced folate carrier. In addition, B cells are FR-negative and cannot respond to EC2319 ± C0923 [48]. Further studies in relation to the effect of EC2319 on humoral immune response will be required to understand other potential collateral effects of this FA-AMT conjugate. For example, EC2319 reduction in activated monocyte/macrophages that release cytokine/chemokines could reduce the accumulation of other inflammatory immune cells such as B cells.

Notably, although MTX matching EC2319 dose (MTX 3D) decreased circulating levels of antigen-specific IgG and deposition of rat IgG within the glomeruli in anti-GBM GN, it did not protect from kidney injury at the time the rats were euthanized (day 10 after the induction of the disease).

In this model of crescentic GN is observed a considerable number of macrophages at four hours after the administration of the anti-GBM Ab. Recruitment of macrophages is in part because of the interaction between the Fc portion of anti-GBM antibody and Fcγ receptors (FcγR) expressed on these cells [49,50]. The recognition of the Fc portion of the anti-GBM antibody by FcγR stimulates macrophages to release chemokines/cytokines that attract additional macrophages hence amplifying the inflammatory response and causing kidney injury. In our study, EC2319 reduced macrophage infiltration and attenuated the humoral immune response with decreasing kidney injury. In comparison, EC2319 matching MTX dosing regimen (MTX 3D) also affected the humoral immunity by inhibiting autologous antibody production, but it did not protect from kidney damage. These data suggest a difference in the mechanism of action between EC2319 and MTX. In addition, it is indicated that blocking humoral immune response is not enough to prevent kidney injury in anti-GBM GN at the time the rats were euthanized, probably because macrophage infiltration is observed very early at the onset of the disease, and this inflammatory response is quickly amplified by proinflammatory cytokines/chemokines released by macrophages themselves and/or other glomerular cells.

It is important that the protective effect of inhibition of the autologous phase in anti-GBM GN is observed at late time points, around 4 weeks after the induction of the disease [51,52,53]. Therefore, it is probable that a beneficial effect of MTX (3D) was not detected at the time the rats were euthanized (day 10). Consequently, it might be possible that later in the course of the disease, a protective effect of MTX (3D) by inhibiting the autologous phase might be observed, and even better protection could also be found with EC2319 by blocking both activated macrophages and the autologous phase.

In summary, this study indicates targeting activated macrophages using high-affinity FR-specific ligands to deliver anti-inflammatory agents directly to these activated cells could represent a novel means for targeting chronic inflammatory diseases.

EC2319 targeting of activated monocytes/macrophages is highly FR-specific and likely has fewer side effects than using multiple immunosuppressive treatments. EC2319 exploits the fact that activated macrophages express a functional FRβ to trick macrophages into carrying the means of their own destruction without affecting nonactivated macrophages. In addition, another advantage of EC2319 is that it has a short elimination half-life that is favorable because it minimizes nonspecific tissue exposure without reducing the FR targeting potential [21].

In conclusion, EC2319′s ability to attenuate progressive kidney injury is primarily driven by blocking activated monocyte/macrophage and perhaps has a collateral effect on antigen-specific humoral immunity.

### Limitations of Study

The limitations of this study are that animal models cannot fully reflect the complexity of human diseases. Owing to the difficulty of obtaining relevant clinical samples, we have not been able to directly compare the level of FR expression on activated macrophages in anti-GBM GN in rats with that found in on nephritic kidneys of patients. In addition, because of its molecular properties (size, charge), EC2319 does not meet the common criteria for oral drug delivery; therefore, this route of dosing was not explored.

Another cautious limitation of this study is that circulating serum folate levels in rats are supra-physiological high because of the supplementation of commercial chows. Because high folate levels can act as a competitor for FR binding, rats used in this study were fed a nonsupplemented diet. It is theoretically possible FR levels in resident macrophages may have inadvertently been upregulated in the nephritic rats during the study. Nevertheless, EC2319 treatment was found to be effective in attenuating kidney injury and appeared to be FR-specific since a benign folate ligand (EC0923) efficiently blocked its effect by competing for FR-binding sites.

## Data Availability

Data available upon request.
